# Supplementary material for: Unveiling the Domain-Specific and RAS Isoform-Specific Details of BRAF Regulation
Source: bioRxiv. 2023 Sep 27:2023.04.24.538112. Originally published 2023 Apr 24. Preprint. [Version 2] doi: 10.1101/2023.04.24.538112 (PMC10168249; doi:10.1101/2023.04.24.538112)
Supplement: Supplement 2 [file NIHPP2023.04.24.538112v2-supplement-2.pdf]

bioRxiv preprint doi: https://doi.org/10.1101/2023.04.24.538112; this version posted September 27, 2023. The copyright holder for this preprint (which was not certified by peer review) is the author/funder, who has granted bioRxiv a license to display the preprint in perpetuity. It is made available under aCC-BY 4.0 International license.

Supplementary Figures

Unveiling the Domain-Specific and RAS Isoform-Specific Details of BRAF Regulation

Tarah Trebino[1], Borna Markusic[1,2], Haihan Nan[1,3], Shrhea Banerjee[1], Zhihong Wang[1,4]

[1]Rowan University, 201 Mullica Hill Rd, Glassboro, NJ 08028

[2]Max Planck Institute of Biophysics, Max-von-Laue Straße 3, 60438 Frankfurt am Main, Germany

[3]School of Laboratory Medicine and Life Science, Wenzhou Medical University, Wenzhou, Zhejiang, China 325035

[4]corresponding author (wangz@rowan.edu)

bioRxiv preprint doi: https://doi.org/10.1101/2023.04.24.538112; this version posted September 27, 2023. The copyright holder for this preprint (which was not certified by peer review) is the author/funder, who has granted bioRxiv a license to display the preprint in perpetuity. It is made available under aCC-BY 4.0 International license.

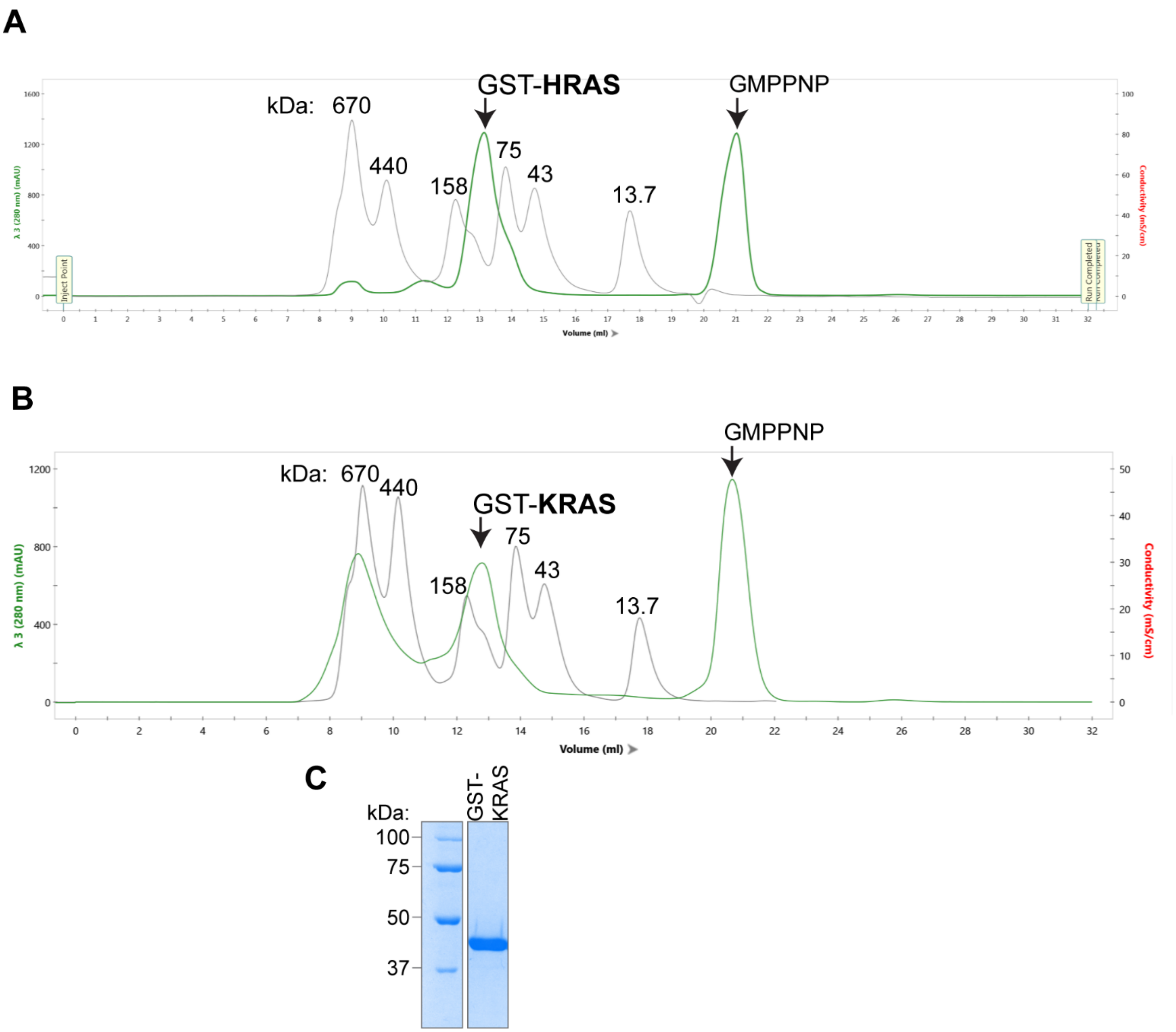

Supplementary Figure 1- SEC of active GST-HRAS and GST-KRAS. (A) GST-HRAS monomer is ~45 kDa and elutes as a dimer at ~90 kDa. HRAS elution profile (green) on a Superdex 200 (Cytiva) overlayed with protein size standard elution profile (gray). (B) GST-KRAS elution profile details the same as GST-HRAS (A). Fractions from ~12-14 mL were collected and concentrated. (C) Coomassie stained gel of GST-KRAS final purification product.

bioRxiv preprint doi: https://doi.org/10.1101/2023.04.24.538112; this version posted September 27, 2023. The copyright holder for this preprint (which was not certified by peer review) is the author/funder, who has granted bioRxiv a license to display the preprint in perpetuity. It is made available under aCC-BY 4.0 International license.

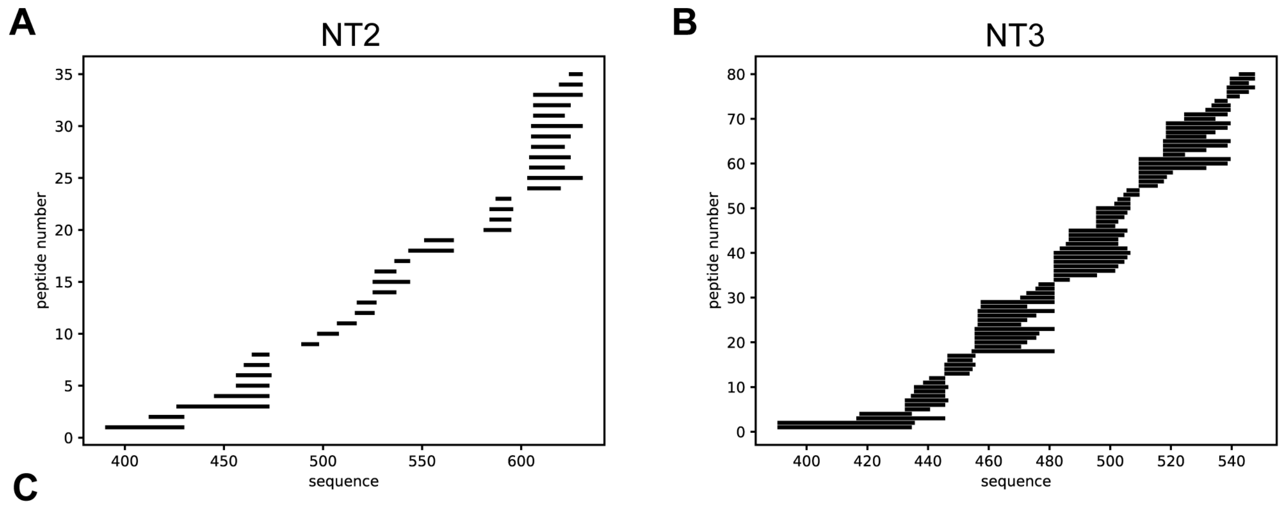

C

NT2 sequence:
1- **MGSSHHHHHHGTKTEEGKLVIWINGDKGYNGLAEVGKKFEKDTGIKVTVEHPDKLEEKFPQ-VAATGDGPDIIFWAHDRFGGYAQSGLLAEITPDKAFQDKLYPFTWDAVRYNGKLIAYPIAVEALSLIYNKDLLPNPPKTWEEIPALDKELKAKGKSALMFNLQEPYFTWPLIAADGGYAFKYENGKYDIKDVGVDNAGAKAGLTFLVDLIKNKHMNADTDYSIAEAAFNKGETAMTINGPWAWSNIDTSKVNYGVTVLPTFKGQPSKPFVGVLSAGINAASPNKELAKEFLENYLLTDEGLEAVNKDKPLGAVALKSYEEELAKDPRIAATMENAQKGEIMPNIPQMSAFWYAVRTAVINAASGRQTVDEAL**KDAQTGTDYDIPTTENLYFQGHMASMTGGQQMGRG**SAALSGGGGGGAEPGQALFNGDMEPEAGAGAGAAASSAADPAIPEEVWNIKQMIKLTQEHIEALLDKFGGEHNPPSIYLEAYEEYTSKLDALQQREQQLLESLGNGTDFSVSSSASMDTVTSSSSSSLSVLPSSLSVFQNPTDVARSNPKSPQKPIVRVFLPNKQRTVVPARCGVTVRDSLKKALMMRGLIPECCAVYRIQDGEKKPIGWDTDISWLTGEELHVEVL** -632

His/MBP tag
BRAF NT2 (a.a. 1-227)

NT3 sequence:
1- **MGSSHHHHHHGTKTEEGKLVIWINGDKGYNGLAEVGKKFEKDTGIKVTVEHPDKLEEKFPQ-VAATGDGPDIIFWAHDRFGGYAQSGLLAEITPDKAFQDKLYPFTWDAVRYNGKLIAYPIAVEALSLIYNKDLLPNPPKTWEEIPALDKELKAKGKSALMFNLQEPYFTWPLIAADGGYAFKYENGKYDIKDVGVDNAGAKAGLTFLVDLIKNKHMNADTDYSIAEAAFNKGETAMTINGPWAWSNIDTSKVNYGVTVLPTFKGQPSKPFVGVLSAGINAASPNKELAKEFLENYLLTDEGLEAVNKDKPLGAVALKSYEEELAKDPRIAATMENAQKGEIMPNIPQMSAFWYAVRTAVINAASGRQTVDEAL**KDAQTGTDYDIPTTENLYFQGHMASMTGGQQMGRGS**SPQKPIVRVFLPNKQRTVVPARCGVTVRDSLKKALMMRGLIPECCAVYRIQDGEKKPIGWDTDISWLTGEELHVEVLENVPLTTHNFVRKTFFTLAFCDFCRKLLFQGFRCQTCGYKFHQRCSTEVPLMCVNYDQLDL** -543

His/MBP tag
BRAF NT3 (a.a. 151-288)

Supplementary Figure 2. Stripe Plots for peptides identified in BRAF-NT2 (a) and NT3 (b). Peptide coverage shown begins at the start of BRAF-NT2 or NT3 (not shown-coverage for MBP-tag N-terminal to the BRAF sequence). (C) Protein sequences used in MS analysis.

bioRxiv preprint doi: https://doi.org/10.1101/2023.04.24.538112; this version posted September 27, 2023. The copyright holder for this preprint (which was not certified by peer review) is the author/funder, who has granted bioRxiv a license to display the preprint in perpetuity. It is made available under aCC-BY 4.0 International license.

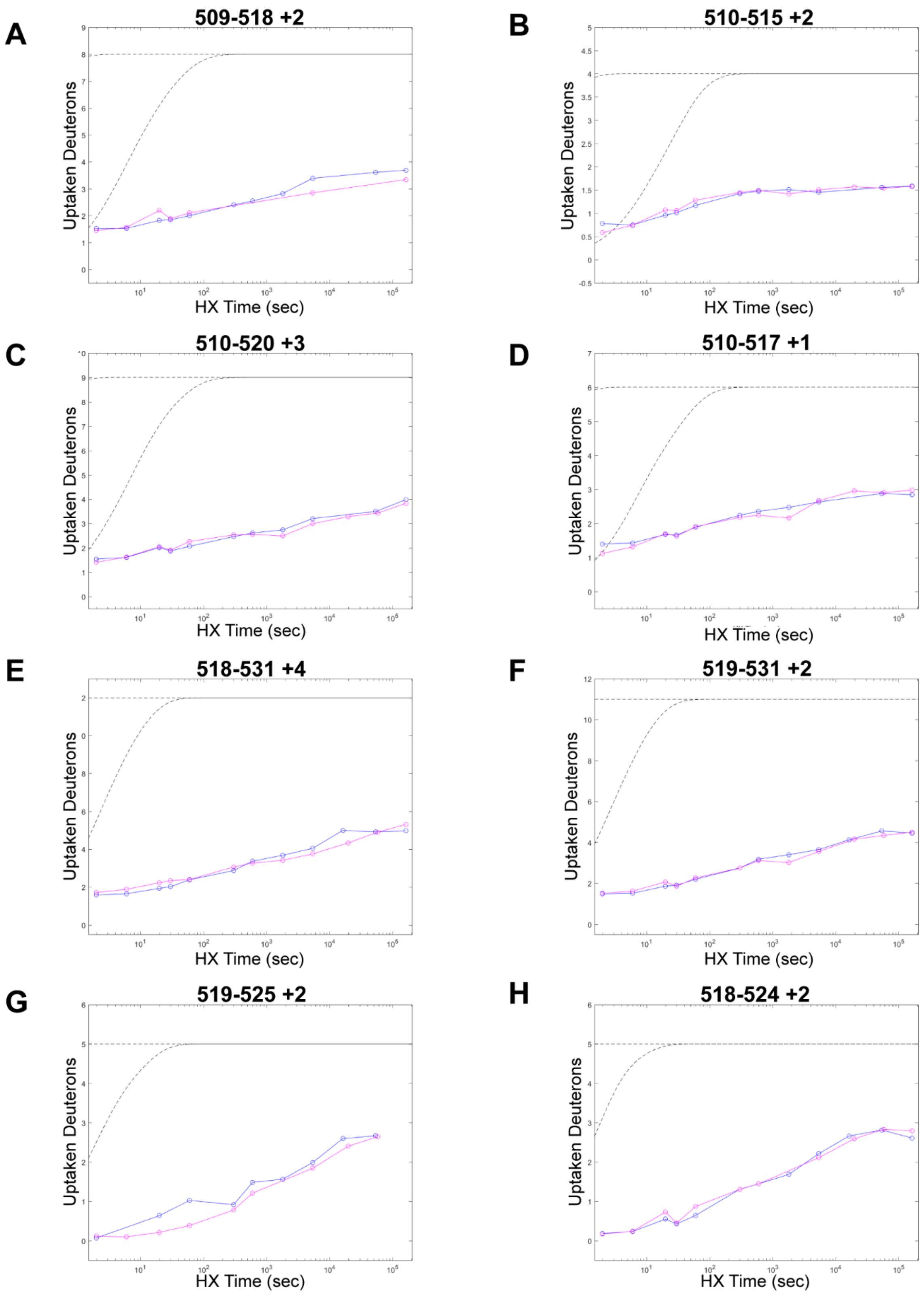

Supplementary Figure 3. Peptides from NT3 in the CRD region. Plots on left represent peptides that could have slowed deuterium uptake. Plots on right represent peptides in

bioRxiv preprint doi: https://doi.org/10.1101/2023.04.24.538112; this version posted September 27, 2023. The copyright holder for this preprint (which was not certified by peer review) is the author/funder, who has granted bioRxiv a license to display the preprint in perpetuity. It is made available under aCC-BY 4.0 International license.

the same region that show essentially no change. Blue= NT3-apo; Magenta= NT3+HRAS. BRAF residues 232-284 (CRD)= peptide residues 491-543. Gray dotted lines represent the theoretical exchange behavior for specified peptide that is fully unstructured (top) or for specified peptide with a uniform protection factor (fraction of time the residue is involved in protecting the H-bond) of 100 (lower).

bioRxiv preprint doi: https://doi.org/10.1101/2023.04.24.538112; this version posted September 27, 2023. The copyright holder for this preprint (which was not certified by peer review) is the author/funder, who has granted bioRxiv a license to display the preprint in perpetuity. It is made available under aCC-BY 4.0 International license.

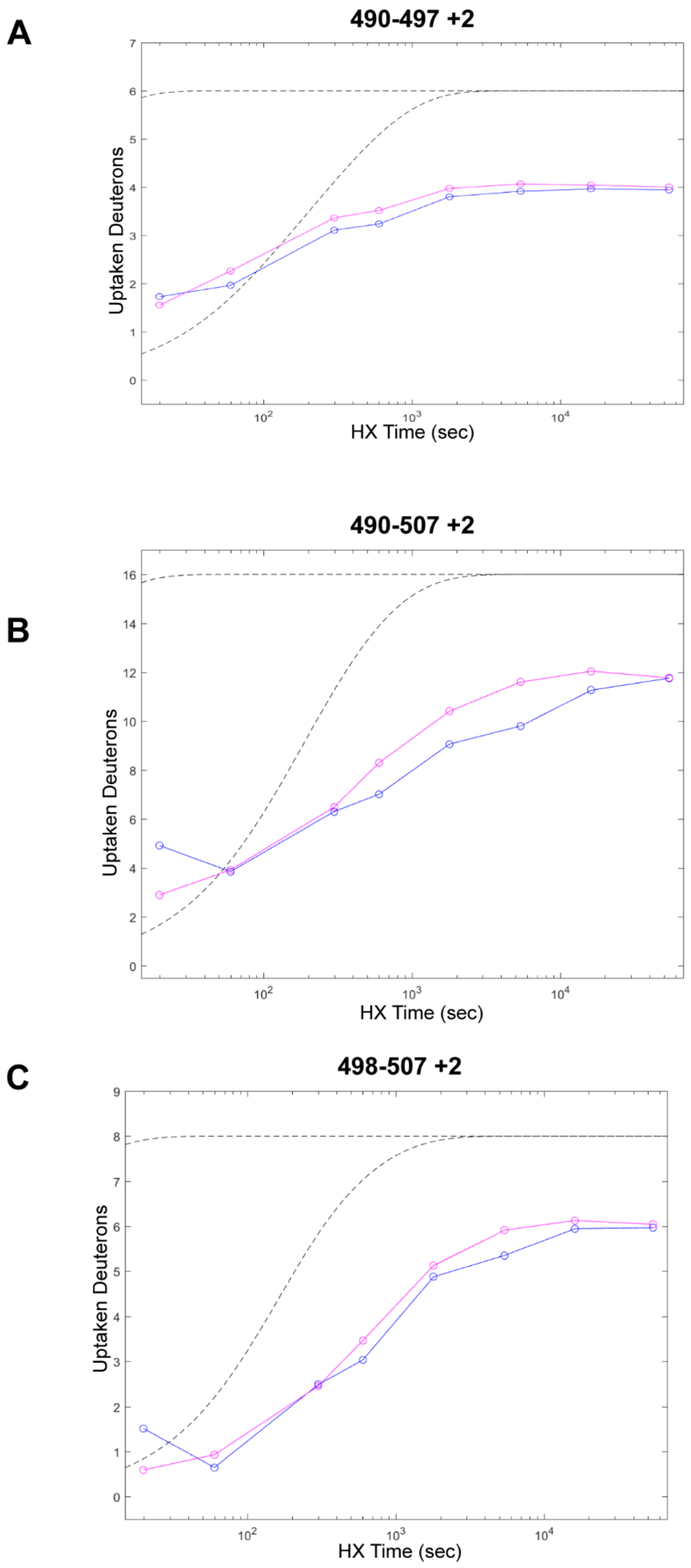

bioRxiv preprint doi: https://doi.org/10.1101/2023.04.24.538112; this version posted September 27, 2023. The copyright holder for this preprint (which was not certified by peer review) is the author/funder, who has granted bioRxiv a license to display the preprint in perpetuity. It is made available under aCC-BY 4.0 International license.

Supplementary Figure 4. Peptides from NT2 in the BSR region (aa. 82-99). Three representative peptide plots that have increased deuterium uptake. Blue= NT2-apo; Magenta= NT2+HRAS. BRAF residues 82-99 correspond with peptide residues 490-507. Gray dotted lines represent the theoretical exchange behavior for specified peptide that is fully unstructured (top) or for specified peptide with a uniform protection factor (fraction of time the residue is involved in protecting the H-bond) of 100 (lower).

bioRxiv preprint doi: https://doi.org/10.1101/2023.04.24.538112; this version posted September 27, 2023. The copyright holder for this preprint (which was not certified by peer review) is the author/funder, who has granted bioRxiv a license to display the preprint in perpetuity. It is made available under aCC-BY 4.0 International license.

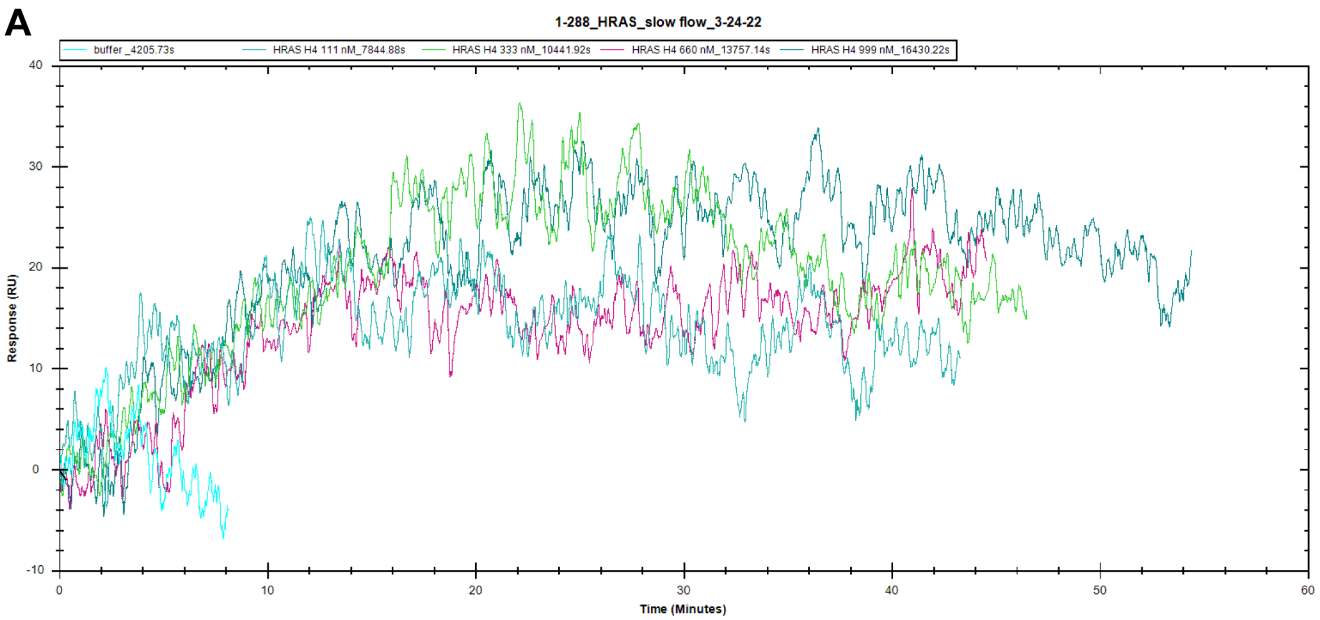

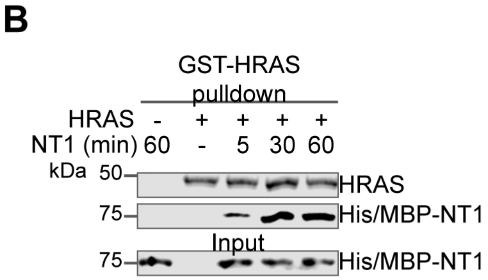

Supplementary Figure 5. HRAS-NT1 OpenSPR shows slow association. (A) OpenSPR injections of HRAS (111, 333, 660, and 999 nM) at 5 µl/min over His/MBP-NT1 immobilized on an NTA sensor. (B) Western blot of GST-HRAS on glutathione resin and NT1 binding through pulldown assay. HRAS was first added to resin for 1 hour. After washing to remove unbound HRAS, NT1 was added in a 1:1 molar ratio and incubated at 4 °C for 5, 30, and 60 minutes. HRAS was probed with GST antibody and NT1 with His antibody.

bioRxiv preprint doi: https://doi.org/10.1101/2023.04.24.538112; this version posted September 27, 2023. The copyright holder for this preprint (which was not certified by peer review) is the author/funder, who has granted bioRxiv a license to display the preprint in perpetuity. It is made available under aCC-BY 4.0 International license.

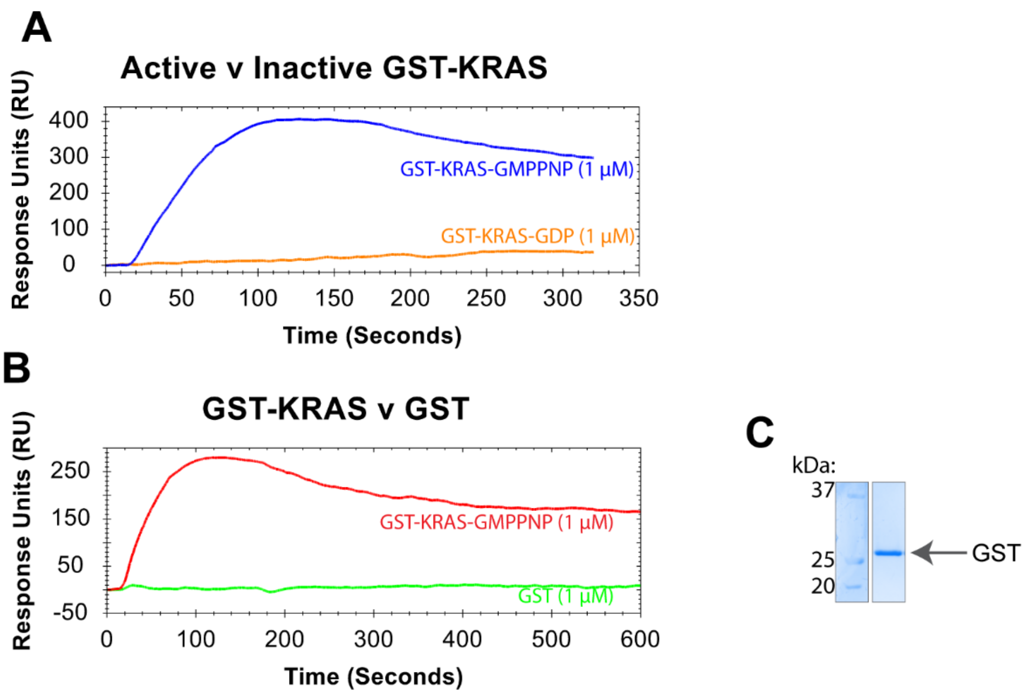

Supplementary Figure 6 (A) Binding curves of 1 μM GST-KRAS (active/GMPPNP= blue; inactive/GDP= orange) flowed over NTA-immobilized BRAF-NT2 at 30 μl/min. (B) Binding curves of 1 μM GST-KRAS (active/GMPPNP-loaded= red) and purified GST (green) flowed over NTA-immobilized BRAF-NT2 at 30 μl/min. (C) Coomassie stained gel of GST protein, purified following the same protocol as GST-HRAS.

bioRxiv preprint doi: https://doi.org/10.1101/2023.04.24.538112; this version posted September 27, 2023. The copyright holder for this preprint (which was not certified by peer review) is the author/funder, who has granted bioRxiv a license to display the preprint in perpetuity. It is made available under aCC-BY 4.0 International license.

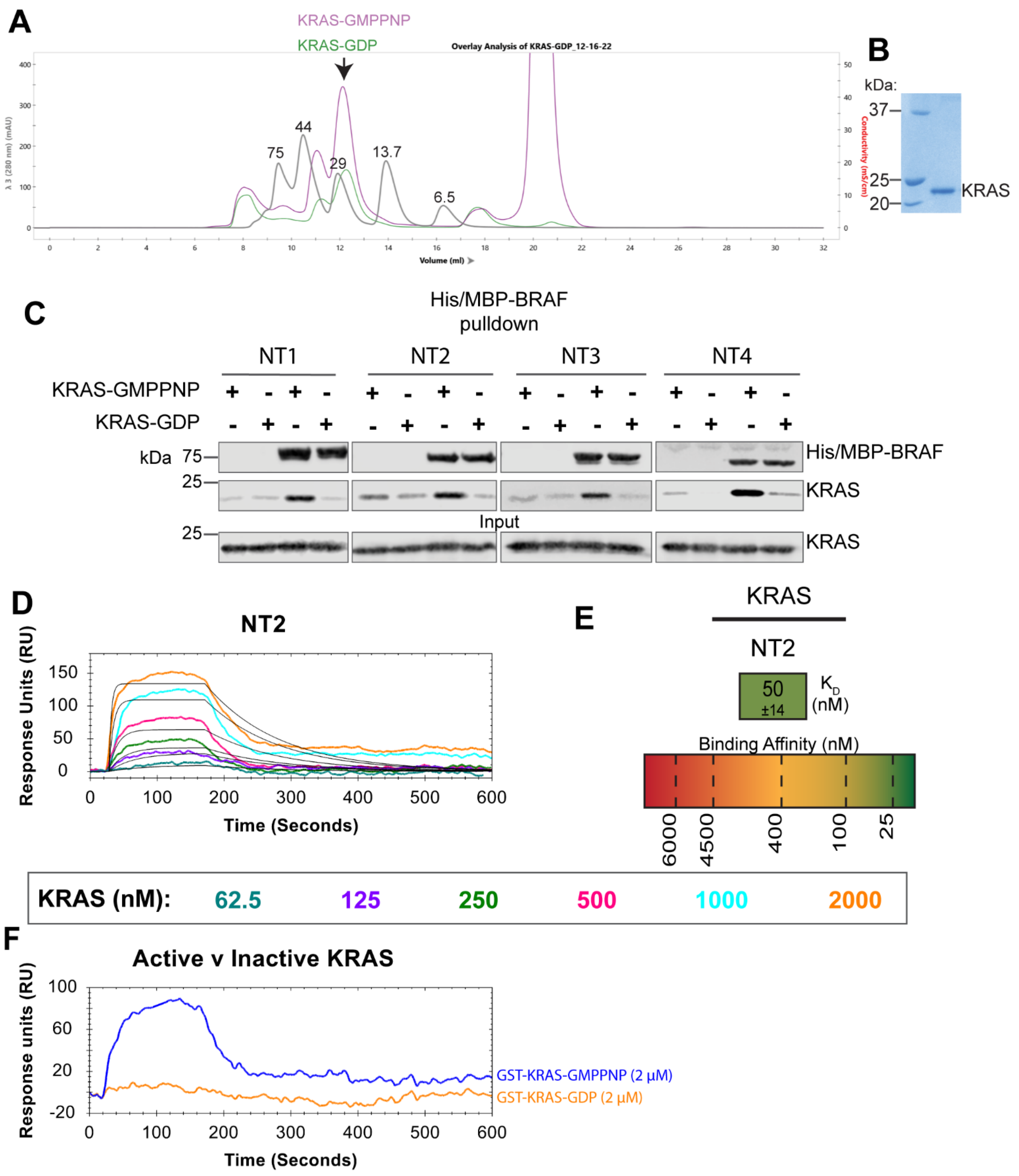

Supplementary Figure 7. Characterizing untagged KRAS (A) Untagged KRAS is displayed as a monomer with a molecular weight of ~22 kDa including N-terminal linker residues. KRAS-GMPPNP (purple) and KRAS-GDP (green) elution profile on a

bioRxiv preprint doi: https://doi.org/10.1101/2023.04.24.538112; this version posted September 27, 2023. The copyright holder for this preprint (which was not certified by peer review) is the author/funder, who has granted bioRxiv a license to display the preprint in perpetuity. It is made available under aCC-BY 4.0 International license.

Superdex 75 (Cytiva) overlayed with protein size standard elution profile (gray). (B) Coomassie stained gel of untagged KRAS final purification product. (C) Western blot of purified KRAS binding to His/MBP-NT1-4 on amylose resin in a pulldown assay. Representative of 3 independent experiments with similar results. (D) OpenSPR binding curves of KRAS flowed over NT2 and the best fit curves produced from a 1:1 fitting model kinetic evaluation. Representative of 2 independent experiments with similar results each. KRAS was flowed over at 30 μL/min (20 mM HEPES pH 7.4, 150 mM NaCl, 0.05% Tween-20, 1% w:v BSA) for 10 mins at increasing concentrations (62.5, 125, 250, 500, 1000, 2000 nM). (E) Diagram of the average binding constant (KD) ± standard deviation from independent OpenSPR experiments of KRAS flowed over immobilized NT2. (F) Binding curves of 2 μM KRAS (active/GMPPNP= blue; inactive/GDP= orange) flowed over NTA-immobilized BRAF-NT2 at 30 μl/min.

bioRxiv preprint doi: https://doi.org/10.1101/2023.04.24.538112; this version posted September 27, 2023. The copyright holder for this preprint (which was not certified by peer review) is the author/funder, who has granted bioRxiv a license to display the preprint in perpetuity. It is made available under aCC-BY 4.0 International license.

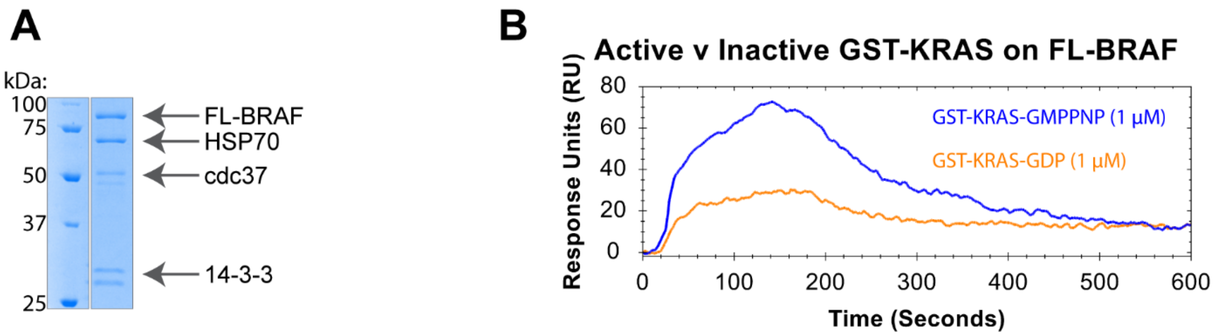

Supplementary Figure 8 (A) Coomassie stained gel of purified recombinant full-length (FL) BRAF with copurified chaperone proteins. (B) Binding curves of 1 µM GST-KRAS (active/GMPPNP = blue; inactive/GDP= orange) flowed over NTA-immobilized FL-BRAF at 30 µl/min.

bioRxiv preprint doi: https://doi.org/10.1101/2023.04.24.538112; this version posted September 27, 2023. The copyright holder for this preprint (which was not certified by peer review) is the author/funder, who has granted bioRxiv a license to display the preprint in perpetuity. It is made available under aCC-BY 4.0 International license.

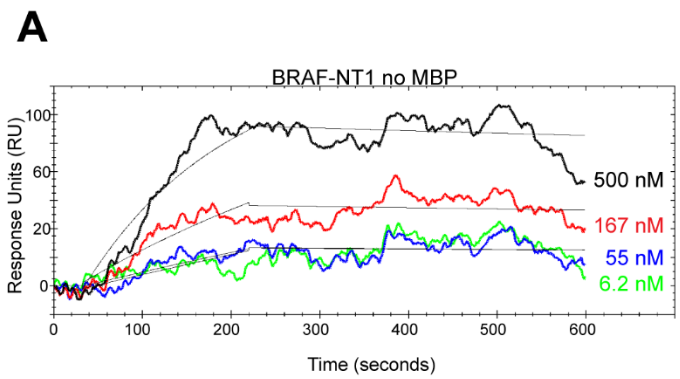

Supplementary Figure 9 (A) OpenSPR binding curves of BRAF-NT1 without MBP flowed over immobilized BRAF-KD at 30 μl/min in increasing concentrations. Best fit curves (black) are produced from a 1:1 fitting model kinetic evaluation.
