# Supplementary material for: Unveiling the Domain-Specific and RAS Isoform-Specific Details of BRAF Regulation
Source: bioRxiv. 2023 Apr 24:2023.04.24.538112. Preprint. [Version 1] doi: 10.1101/2023.04.24.538112 (PMC10168249; doi:10.1101/2023.04.24.538112)
Supplement: Supplementary file 1 [file NIHPP2023.04.24.538112v1-supplement-1.pdf]

bioRxiv preprint doi: https://doi.org/10.1101/2023.04.24.538112; this version posted April 24, 2023. The copyright holder for this preprint (which was not certified by peer review) is the author/funder, who has granted bioRxiv a license to display the preprint in perpetuity. It is made available under aCC-BY 4.0 International license.

Supplementary Figures:

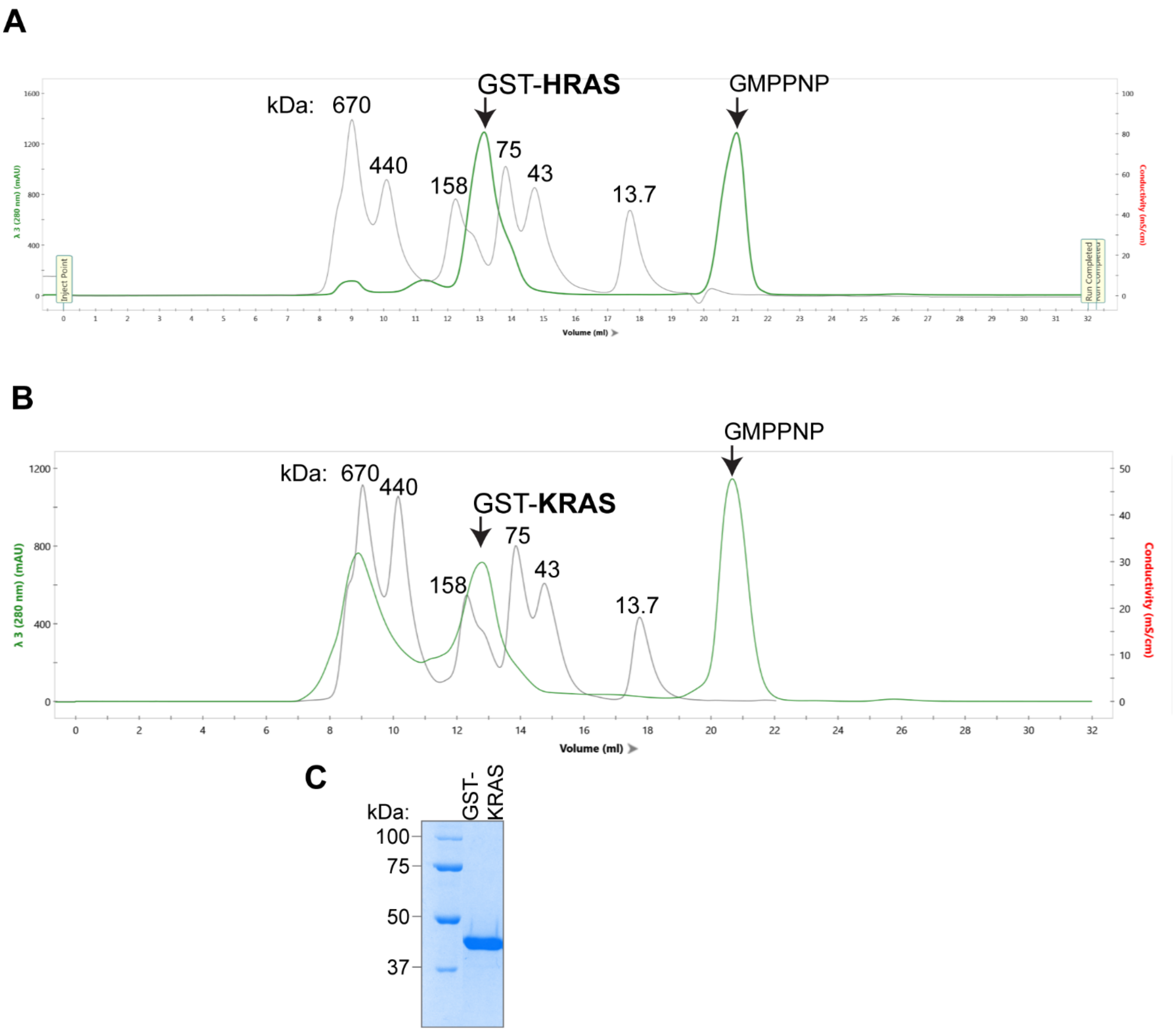

Supplementary Figure 1- SEC of active GST-HRAS and GST-KRAS. (A) GST-HRAS monomer is ~45 kDa and elutes as a dimer at ~90 kDa. HRAS elution profile (green) on a Superdex 200 (Cytiva) overlayed with protein size standard elution profile (gray). (B) GST-KRAS elution profile details the same as GST-HRAS (A). Fractions from ~12-14 mL were collected and concentrated. (C) Coomassie stained gel of GST-KRAS final purification product.

bioRxiv preprint doi: https://doi.org/10.1101/2023.04.24.538112; this version posted April 24, 2023. The copyright holder for this preprint (which was not certified by peer review) is the author/funder, who has granted bioRxiv a license to display the preprint in perpetuity. It is made available under aCC-BY 4.0 International license.

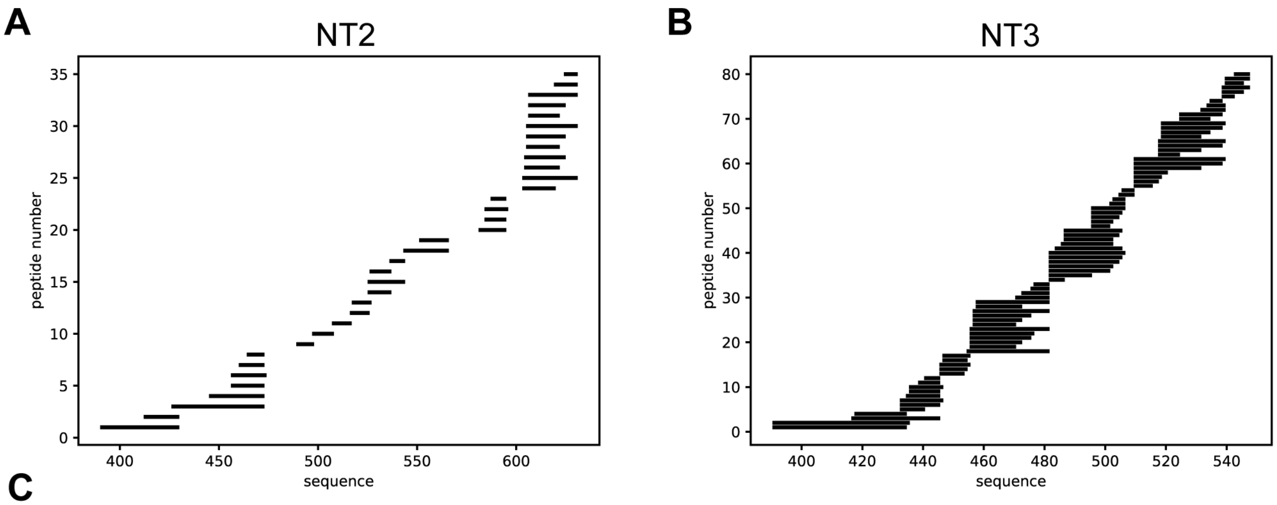

C

NT2 sequence:

1- **MGSSHHHHHHGTKTEEGKLVIWINGDKGYNGLAEVGKKFEKDTGIKVTVEHPDKLEEKFPQ-VAATGDGPDIIFWAHDRFGGYAQSGLLAEITPDKAFQDKLYPFTWDAVRYNGKLIAYPIAVEALSLIYNKDLLPNPPKTWEEIPALDKELKAKGKSALMFNLQEPYFTWPLIAADGGYAFKYENGKYDIKDVGVDNAGAKAGLTFLVDLIKNKHMNADTDYSIAEAAFNKGETAMTINGPWAWSNIDTSKVNYGVTVLPTFKGQPSKPFVGVLSAGINAASPNKELAKEFLENYLLTDEGLEAVNKDKPLGAVALKSYEEELAKDPRIAATMENAQKGEIMPNIPQMSAFWYAVRTAVINAASGRQTVDEAL**KDAQTGTDYDIPTTENLYFQGHMASMTGGQQMGRG**SAALSGGGGGGAEPGQALFNGDMEPEAGAGAGAAASSAADPAIPEEVWNIKQMIKLTQEHIEALLDKFGGEHNPPSIYLEAYEEYTSKLDALQQREQQLLESLGNGTDFSVSSSASMDTVTSSSSSSLSVLPSSLSVFQNPTDVARSNPKSPQKPIVRVFLPNKQRTVVPARCGVTVRDSLKKALMMRGLIPECCAVYRIQDGEKKPIGWDTDISWLTGEELHVEVL** -632

His/MBP tag
BRAF NT2 (a.a. 1-227)

NT3 sequence:

1- **MGSSHHHHHHGTKTEEGKLVIWINGDKGYNGLAEVGKKFEKDTGIKVTVEHPDKLEEKFPQ-VAATGDGPDIIFWAHDRFGGYAQSGLLAEITPDKAFQDKLYPFTWDAVRYNGKLIAYPIAVEALSLIYNKDLLPNPPKTWEEIPALDKELKAKGKSALMFNLQEPYFTWPLIAADGGYAFKYENGKYDIKDVGVDNAGAKAGLTFLVDLIKNKHMNADTDYSIAEAAFNKGETAMTINGPWAWSNIDTSKVNYGVTVLPTFKGQPSKPFVGVLSAGINAASPNKELAKEFLENYLLTDEGLEAVNKDKPLGAVALKSYEEELAKDPRIAATMENAQKGEIMPNIPQMSAFWYAVRTAVINAASGRQTVDEAL**KDAQTGTDYDIPTTENLYFQGHMASMTGGQQMGRGS**SPQKPIVRVFLPNKQRTVVPARCGVTVRDSLKKALMMRGLIPECCAVYRIQDGEKKPIGWDTDISWLTGEELHVEVLENVPLTTHNFVRKTFFTLAFCDFCRKLLFQGFRCQTCGYKFHQRCSTEVPLMCVNYDQLDL** -543

His/MBP tag
BRAF NT3 (a.a. 151-288)

Supplementary Figure 2. Stripe Plots for peptides identified in BRAF-NT2 (a) and NT3 (b). Peptide coverage shown begins at the start of BRAF-NT2 or NT3 (not shown: coverage for MBP-tag N-terminal to the BRAF sequence). (C) Protein sequences used in MS analysis.

bioRxiv preprint doi: https://doi.org/10.1101/2023.04.24.538112; this version posted April 24, 2023. The copyright holder for this preprint (which was not certified by peer review) is the author/funder, who has granted bioRxiv a license to display the preprint in perpetuity. It is made available under aCC-BY 4.0 International license.

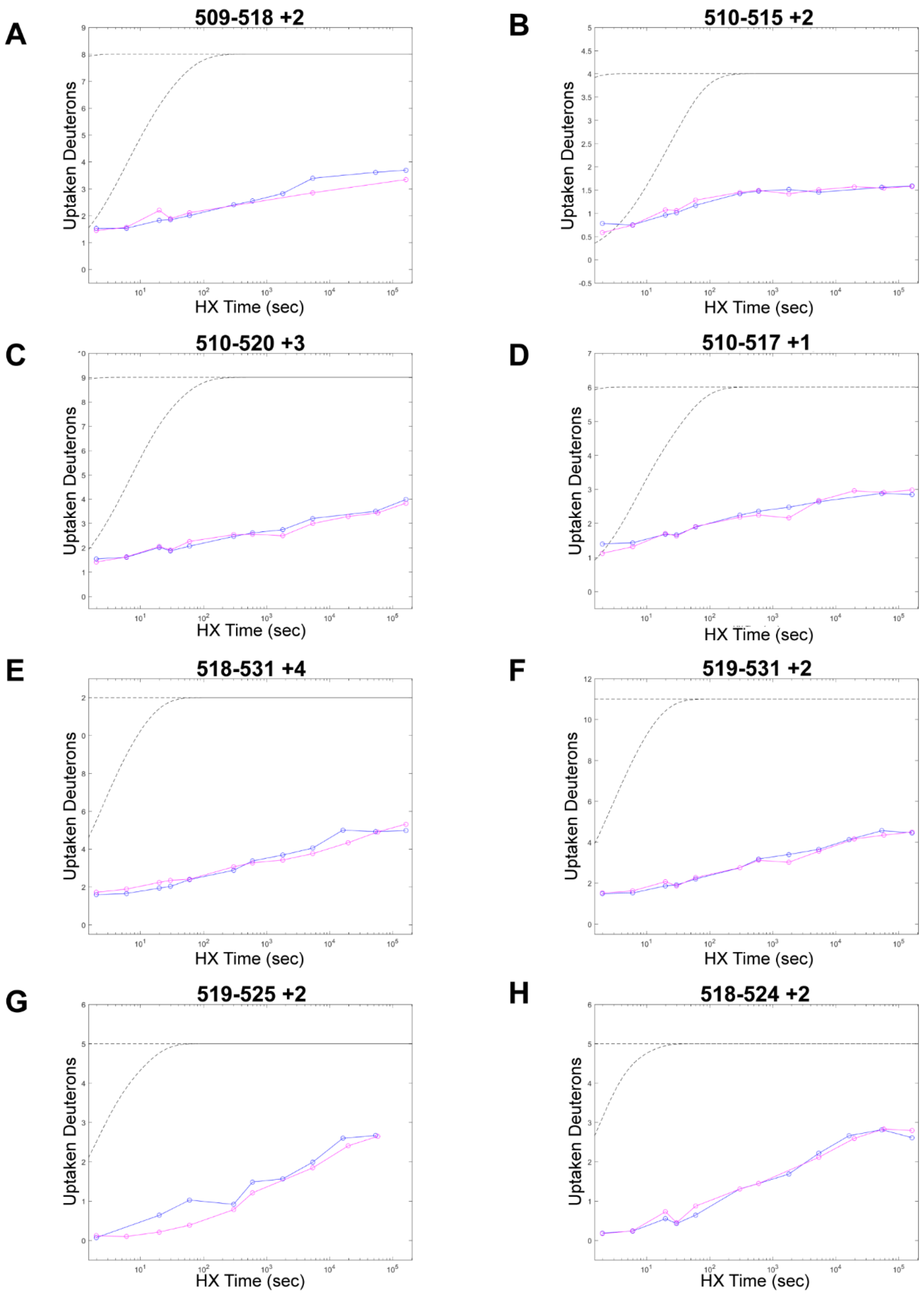

Supplementary Figure 3. Peptides from NT3 in the CRD region. Plots on left represent peptides that could have slowed deuterium uptake. Plots on right represent peptides in the same region

bioRxiv preprint doi: https://doi.org/10.1101/2023.04.24.538112; this version posted April 24, 2023. The copyright holder for this preprint (which was not certified by peer review) is the author/funder, who has granted bioRxiv a license to display the preprint in perpetuity. It is made available under aCC-BY 4.0 International license.

that show essentially no change. Blue= NT3-apo; Magenta= NT3+HRAS. BRAF residues 232-284 (CRD)= peptide residues 491-543.

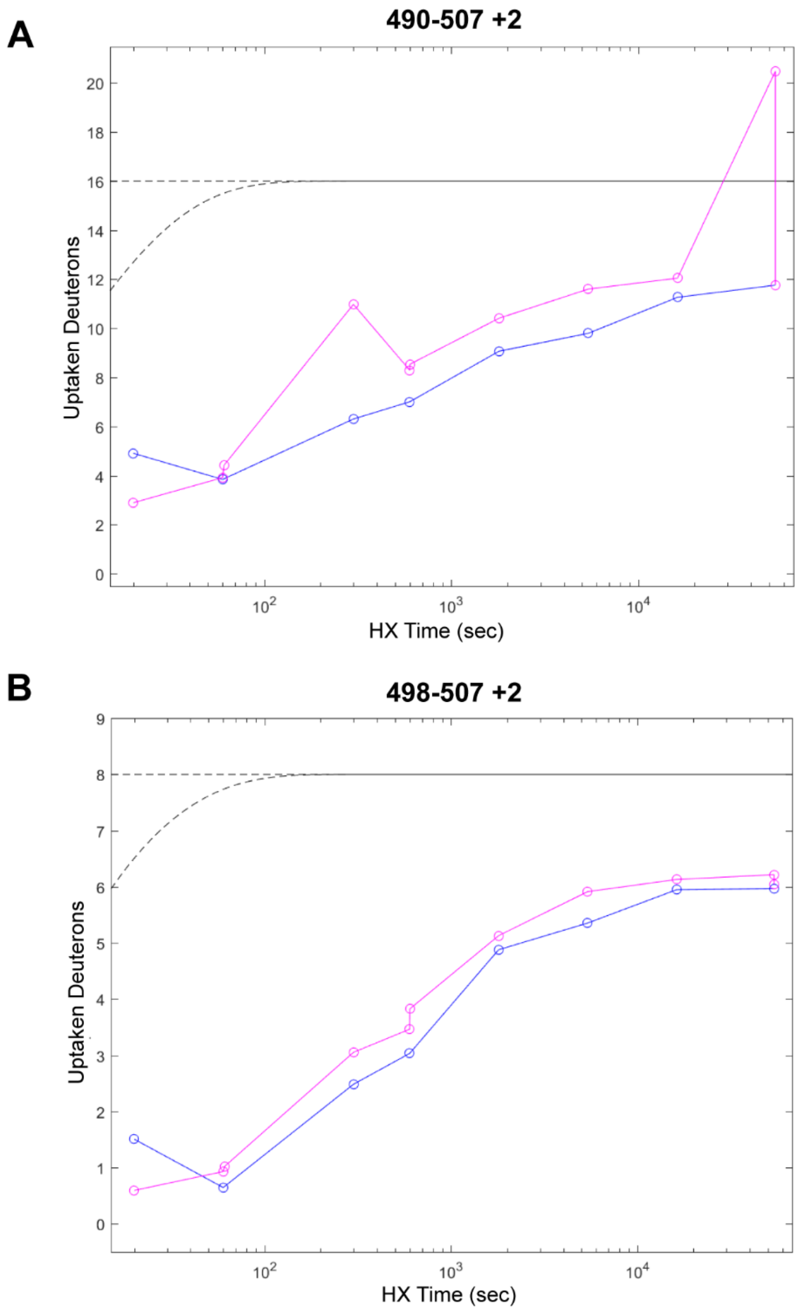

Supplementary Figure 4. Peptides from NT2 in the BSR region (aa. 82-99). Two representative peptide plots that have increased deuterium uptake. Blue= NT2-apo; Magenta= NT2+HRAS. BRAF residues 82-99 correspond with peptide residues 490-507.

bioRxiv preprint doi: https://doi.org/10.1101/2023.04.24.538112; this version posted April 24, 2023. The copyright holder for this preprint (which was not certified by peer review) is the author/funder, who has granted bioRxiv a license to display the preprint in perpetuity. It is made available under aCC-BY 4.0 International license.

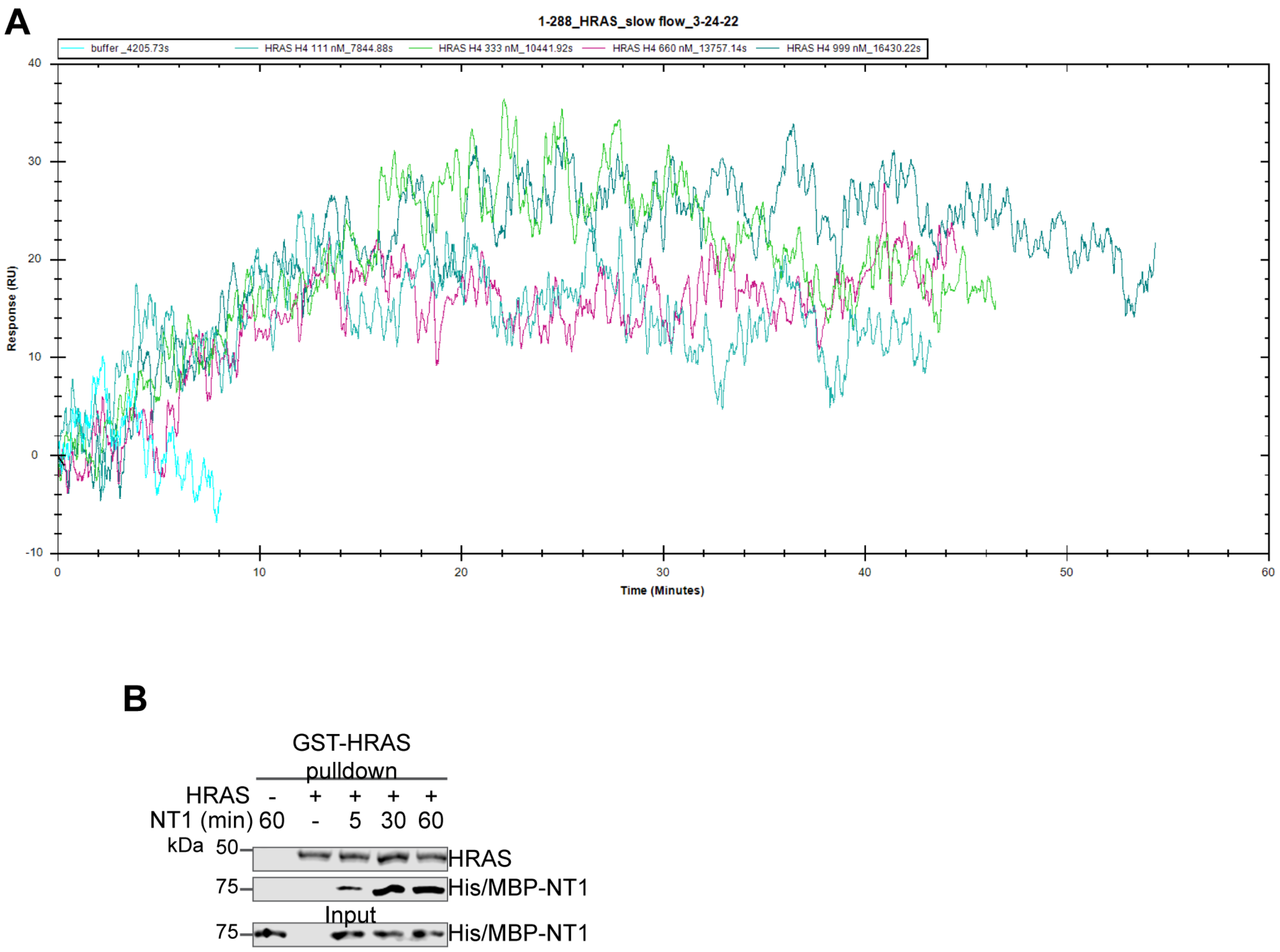

Supplementary Figure 5. HRAS-NT1 SPR shows slow association. (A) OpenSPR injections of HRAS (111, 333, 660, and 999 nM) at 5 ul/min over His/MBP-NT1 immobilized on an NTA sensor. (B) Western blot of GST-HRAS on glutathione resin and NT1 binding through pulldown assay. HRAS was first added to resin for 1 hour. After washing to remove unbound HRAS, NT1 was added in a 1:1 molar ratio and incubated at 4 °C for 5, 30, and 60 minutes. HRAS was probed with GST antibody and NT1 with His antibody.
